# E-cadherin variants associated with oral facial clefts trigger aberrant cell motility in a REG1A-dependent manner

**DOI:** 10.1186/s12964-024-01532-x

**Published:** 2024-02-27

**Authors:** Joana Pereira, Soraia Melo, Rui M. Ferreira, Patrícia Carneiro, Vítor Yang, André F. Maia, João Carvalho, Ceu Figueiredo, José Carlos Machado, Eurico Morais-de-Sá, Raquel Seruca, Joana Figueiredo

**Affiliations:** 1 i3S - Instituto de Investigação e Inovação em Saúde, Universidade Do Porto, Rua Alfredo Allen, 208, Porto, 4200-135 Portugal; 2 IPATIMUP - Institute of Molecular Pathology and Immunology of Porto University, Porto, Portugal; 3 Faculty of Medicine, University of Porto, Porto, Portugal; 4 IBMC - Institute for Molecular and Cell Biology, University of Porto, Porto, Portugal; 5 ICBAS - Institute of Biomedical Sciences Abel Salazar, University of Porto, Porto, Portugal; 6 CFisUC, Department of Physics, University of Coimbra, Coimbra, Portugal

**Keywords:** E-cadherin, Oral facial clefts, Hereditary diffuse gastric cancer, Cell migration, Extracellular matrix, REG1A

## Abstract

**Background:**

Germline mutations of E-cadherin contribute to hereditary diffuse gastric cancer (HDGC) and congenital malformations, such as oral facial clefts (OFC). However, the molecular mechanisms through which E-cadherin loss-of-function triggers distinct clinical outcomes remain unknown. We postulate that E-cadherin-mediated disorders result from abnormal interactions with the extracellular matrix and consequent aberrant intracellular signalling, affecting the coordination of cell migration.

**Methods:**

Herein, we developed *in vivo* and *in vitro* models of E-cadherin mutants associated with either OFC or HDGC. Using a Drosophila approach, we addressed the impact of the different variants in cell morphology and migration ability. By combining gap closure migration assays and time-lapse microscopy, we further investigated the migration pattern of cells expressing OFC or HDGC variants. The adhesion profile of the variants was evaluated using high-throughput ECM arrays, whereas RNA sequencing technology was explored for identification of genes involved in aberrant cell motility.

**Results:**

We have demonstrated that cells expressing OFC variants exhibit an excessive motility performance and irregular leading edges, which prevent the coordinated movement of the epithelial monolayer. Importantly, we found that OFC variants promote cell adhesion to a wider variety of extracellular matrices than HDGC variants, suggesting higher plasticity in response to different microenvironments. We unveiled a distinct transcriptomic profile in the OFC setting and pinpointed REG1A as a putative regulator of this outcome. Consistent with this, specific RNAi-mediated inhibition of REG1A shifted the migration pattern of OFC expressing cells, leading to slower wound closure with coordinated leading edges.

**Conclusions:**

We provide evidence that E-cadherin variants associated with OFC activate aberrant signalling pathways that support dynamic rearrangements of cells towards improved adaptability to the microenvironment. This proficiency results in abnormal tissue shaping and movement, possibly underlying the development of orofacial malformations.

**Graphical Abstract:**

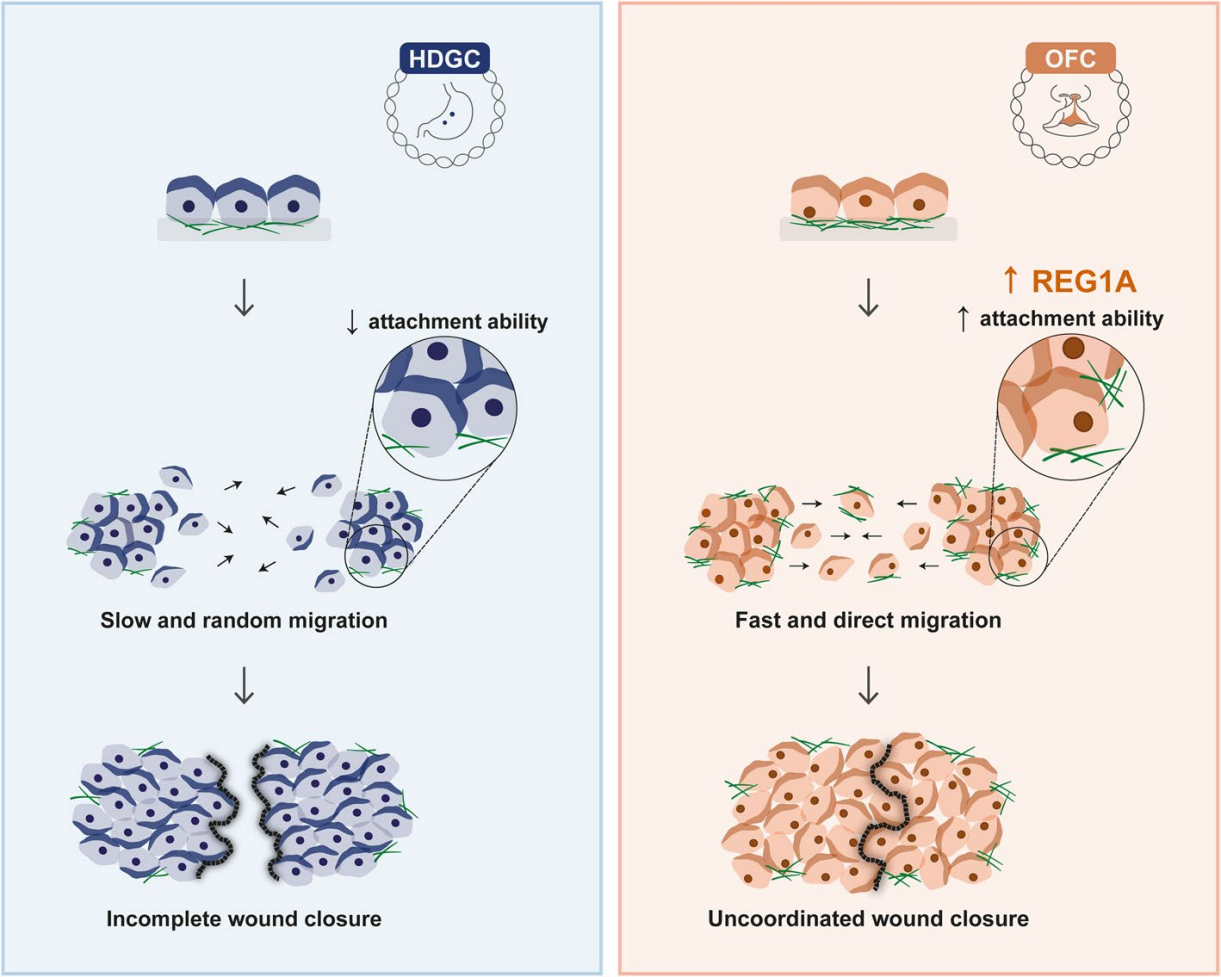

**Supplementary Information:**

The online version contains supplementary material available at 10.1186/s12964-024-01532-x.

## Background

Over the last decades, the role of *CDH1* in cancer aetiology has been extensively investigated. It is well established that *CDH1* pathogenic variants are the cause of hereditary diffuse gastric cancer (HDGC), which is defined by family aggregation of diffuse gastric cancer and lobular breast cancer [1, 2]. In this syndrome, germline mutation carriers develop highly invasive cancers characterized by diffuse spreading of isolated neoplastic cells at the very early stages of disease [2, 3]. Interestingly, congenital malformations have also arisen as a clinical phenotype associated with *CDH1* variants [4]. In particular, oral facial clefts (OFC), such as cleft lip with or without cleft palate (CL/P), or cleft palate alone (CP) have been described both in HDGC family members and as independent disorders associated with *CDH1* genetic alterations [5, 6]. OFC are among the most prevalent birth defects, with an incidence of 1 in 700 newborns worldwide [7]. Affected individuals require comprehensive care as they experience difficulties with feeding, speech, hearing, as well as dental issues and mental health problems [8, 9].

Given that *CDH1* encodes E-cadherin—a critical cell–cell adhesion molecule for tissue function and integrity—it is not unexpected that its disruption may lead to defects during embryonic development and cancer [10, 11]. In fact, correct fusion of the palate is dependent on a complex sequence of processes, such as cell migration, growth, differentiation and apoptosis, coordinated by cell–cell interactions and molecular regulators [12]. In line with this, high levels of E-cadherin expression are detected during critical phases of lip and palate development, including in the fourth and fifth week of human embryogenesis at the frontonasal prominence, and in the sixth week at the lateral and medial nasal prominences [5].

Despite the progress in OFC genetics, the mechanistic effects mediated by E-cadherin that are involved in lip and palate abnormal closure are unknown. In this study, we have addressed the hypothesis that E-cadherin-mediated disorders result from abnormal cell interactions with the extracellular matrix (ECM), and consequent aberrant intracellular signalling, which impact the coordination of epithelial migration. For that purpose, we have explored E-cadherin variants affecting the same nucleotide, although originating distinct clinical outcomes. Taking advantage of *in vivo* and *in vitro* approaches, we investigated unique migratory and adhesive features, as well as transcriptomic profiles generated by HDGC and OFC mutants. Our results demonstrated, for the first time, that variants associated with OFC induce an atypical migration performance and increased cell plasticity in response to different microenvironments. These abilities occur upon activation of a specific molecular program, in which Regenerating Protein 1 α (REG1A) acts as a potential regulator of lip/palate closure.

## Methods

### Plasmid construction

E-cadherin variants D254H (c.760G > C) and P373L (c.1118C > T) found in the HDGC context, and D254N (c.760G > A) and P373R (c.1118C > G) associated with OFC were generated by site-directed mutagenesis in the hCDH1 pIRES2-EGFP vector (Clontech, Takara Bio, Shiga, Japan). For assays involving stable transfection, variants were induced in the entry vector CDH1pENTR 221 (Clone ID: IOH46767, Invitrogen, Grand Island, NY, USA) and subcloned into the pEF6/Myc-His vector (Invitrogen) by LR recombination, as previously described [13]. Direct sequencing was used to verify all cloning vectors, and the corresponding empty vector (Mock) was used as a control.

### Generation of transgenic flies

A transgenic *Drosophila* model based on the Gal4/UAS system was used to target tissue-specific gene expression. The plasmids encoding the D254H, P373L, D254N, and P373R mutant forms in the pENTR backbone were subcloned onto the pPW-attB destination vector (produced in [14]) using LR clonase II-mediated recombination. The different transgenes were then inserted into the attP40 landing site via PhiC31 site-specific transgenesis (CONGENTO, Consortium for Genetically tractable organisms, Portugal), placing all E-cadherin variants under the same genetic environment of a previously published *CDH1* wild-type version [14]. For specific expression in border cells, we generated flies containing UAS-hE-cad wild-type, UAS-hE-cad D254H, UAS-hE-cad P373L, UAS-hE-cad D254N, or UAS-hE-cad P373R together with UAS-CD8::GFP and *slbo-GAL4* (BDSC, Bloomington Drosophila Stock Center #76,363 Genotype: w[*]; P{w[+ mC] = GAL4-slbo.2.6}16, P{y[+ t7.7] w[+ mC] = 10XUAS-IVS-mCD8::GFP}attP40). Fly food was supplemented with yeast, and flies were incubated for 1 day at 29 °C to boost transgene expression and stimulate ovary development. Overexpression of mCherry (BDSC #35,787, Genotype: y^1^ sc^*^ v^1^ sev^21^; P{UAS-mCherry.VALIUM10}attP2) was used as a control.

### Ovary preparation and F-actin staining

*Drosophila* ovaries were dissected in 0.05% Tween-20 in phosphate-buffered saline (PBS). Fixation was performed with 4% paraformaldehyde in PBS for 30 min. Alexa Fluor 562 phalloidin (ThermoFisher) was used for F-actin staining. Ovaries were washed with 0.05% Tween-20 in PBS and mounted in Vectashield with DAPI (Vector Laboratories). Fixed tissue was imaged using an inverted laser scanning confocal microscope (Leica TCS SP5 II, Leica Microsystems) with a HC PL 20x/NA 0.70 objective, or a HC PL APO CS 63x/Glycerol 1.30 objective, and processed using Leica Application Suite software. Fiji was used to measure the migration index in stage 10 egg chambers. The position of the border cell cluster was normalized to the total distance from the anterior part of the egg chamber to the oocyte. Morphological parameters such as length, width, height, sphericity, and surface area of border cells were also analysed. Border cell cluster segmentation was achieved using ilastik 1.3.0 [15], whereas 3D representation was obtained through Imaris software (version 10.0.1).

### Cell Culture and transfection

CHO (Chinese Hamster Ovary, ATCC number CCL-61) and AGS cells (Gastric Adenocarcinoma, ATCC number CRL-1739) were cultured at 37 °C under 5% CO_2_ humidified air, in α-MEM (Gibco, Invitrogen) and RPMI medium (Gibco, Invitrogen), respectively, supplemented with 10% fetal bovine serum (HyClone) and 1% penicillin/streptomycin (Gibco, Invitrogen). Cell transfection was carried out using Lipofectamine 2000 (Invitrogen), according to the manufacturer’s recommended procedures. For overexpression experiments, we used 1 µg of DNA of vectors encoding the wild-type protein or the D254H, P373L, D254N and P373R variants, as well as the empty vector (Mock). Transfected cells were selected by antibiotic resistance to blasticidin (5 μg/ml; Gibco, Invitrogen). For inhibition assays with small interfering RNA (siRNA), a SMARTpool of 4 different siRNAs targeting REG1A mRNA was purchased from Dharmacon and prepared according to the manufacturer’s instructions. Non-silencing siRNA duplexes (Dharmacon) were used as a negative control. Depletion efficiency was at its maximum at 48 h, using 200 nM siRNA.

### Wound healing assays

A suspension of AGS cells at a concentration of 1 × 10^6^ cells/ml was plated onto a 24-well plate with wound healing inserts (CBA-120, Cell Biolabs), and incubated at 37 °C under 5% CO_2_ humidified air. After 24 h, the inserts were removed and cells were washed with PBS to exclude detached cells. Warm supplemented medium was added to the cells and live imaging was started immediately, maintaining culture conditions. Cells were imaged for 24 h, with 10 min intervals, using a Leica DMI6000 microscope (Leica Microsystems) with a 20x objective coupled with the Leica Application Suite X software (Leica Microsystems).

### Cell migration analysis

Cell motile features were assessed through the “Manual Tracking” plugin from Fiji [16]. Specifically, moving individual cells were traced in image sequences, enabling extraction of XY coordinates, and estimation of velocity and distance covered between two frames. Based on these data, cell trajectories were evaluated and mapped. Travelled distance was determined as the sum of all distances covered during 24 h of cell tracking. Mean-Squared Displacement (MSD) of single cells was quantified using a visual basic macro for Microsoft Excel [17], and diffusion (D) was calculated from the expression *MSD* = *4 D t*, where *t* is time (in hours). Wound closure rate was defined as (A_0_—A_n_)/A_0_ × 100, where A_0_ represents the area of the initial wound and A_n_ represents the area of wound at a particular time point. Regularity of the wound gap was estimated as the dispersion of the distance between the edge and the migration front, in a given time.

### ECM microarray

Cell–matrix attachment ability was determined using a MicroMatrix™ of 36 ECM combinations printed on a hydrogel surface (MicroStem). Upon slide hydration, a suspension of CHO cells at a concentration of 2.5 × 10^5^ cells/ml was seeded on the array slides. Slides were incubated at 37 °C under 5% CO_2_ humidified air, allowing cell adhesion to the spots during 48 h. Cells were washed in PBS and fixed in ice-cold methanol for 20 min. Nuclear staining was achieved with a 1 µg/ml DAPI solution. Slide imaging was performed on an automated microscope IN Cell Analyzer 2000 (GE Healthcare) with a Nikon 10x/0.45NA objective. Nuclei segmentation was accomplished with ilastik 1.3.0 [15] and image quantification with CellProfiler [18]. Data was subsequently used for analysis.

### RNA preparation and sequencing

Total RNA was extracted from AGS cells transfected with E-cadherin variants and the corresponding controls, using the RNeasy Mini Kit (Qiagen) in accordance with manufacturer’s instructions. For quality control of total RNA, RNA integrity number (RIN) and concentration were evaluated through the Agilent 2200 TapeStation System. Next generation RNA-seq analysis was conducted by STABVIDA, Lda (Lisbon, Portugal). cDNA library construction was carried out using Kapa Stranded mRNA Library Preparation Kit, and the generated DNA fragments were sequenced in an lllumina NovaSeq 6000 platform with 150 bp paired-end sequencing reads to achieve a target output of 40 M reads per sample. Raw sequencing data was analysed with CLC Genomics Workbench 12.0.3.

### Gene expression analysis and identification of differentially expressed genes (DEGs)

High-quality sequencing reads were mapped against the reference genome, *Homo sapiens* (hg38), using the following settings: length fraction = 0.8 and similarity fraction = 0.8. Reads shorter than 30 nucleotides were excluded, and thus 96.81% to 97.19% of the total fragments were successfully mapped. Gene expression levels were determined through a Transcripts per Million (TPM) approach. Differential expression analysis between sample groups was carried out, and fold changes were calculated using the Generalized Linear Model (GLM), which corrects for differences in library size and confounding factor effects (edgeR, Bioconductor). DEGs were considered when fold change ≥ 2 or ≤ -2, along with FDR *P*-value ≤ 0.05.

### Functional annotation and enrichment analysis

Functional classification and enrichment of Gene Ontology (GO) terms were determined using DAVID (Database for Annotation, Visualization and Integrated Discovery, version 2021), considering a *P*-value of at least 0.05 [19]. The list of DEGs exclusive from the OFC variant group was used for functional annotation. Fold enrichment of GO terms was examined to identify relevant categories. Additionally, clustering strength of GO terms was assessed using the enrichment score estimated with the DAVID Functional Annotation Clustering tool. GO terms and clusters with a fold enrichment > 1 and an enrichment score > 0.9 were considered for further analysis.

### Western blotting

Protein lysates were prepared using catenin lysis buffer [1% Triton X-100 (Sigma) and 1% IGEPAL CA-630 (Sigma) in PBS] supplemented with protease and phosphatase inhibitor cocktails (Roche and Sigma, respectively). Protein concentration was determined using a Bradford-based assay (Bio-Rad). For analysis of total protein samples, 20-40 µg of protein were eluted in sample buffer, separated in 10% or 15% SDS–polyacrylamide gels (SDS-PAGE), and electroblotted onto Hybond ECL membranes (Amersham Biosciences). Membranes were blocked with 5% non-fat milk or 5% BSA in 0.5% Tween-20 in PBS for 1 h, and immunoblotted with antibodies against E-cadherin (1:1000, Clone HECD1, Invitrogen), REG1A (1:500, Invitrogen) and α-Tubulin (1:10,000, Sigma). The secondary antibodies sheep anti-mouse or donkey anti-rabbit HRP-conjugated (Amersham Biosciences) were then incubated, followed by detection with ECL reagents (Bio-Rad). Protein bands were quantified using the Quantity One 4.6.9 Software (Bio-Rad).

### Immunofluorescence

Cells were cultured on glass coverslips, washed with PBS and fixed in ice-cold methanol for 20 min. Fixed cells were washed in PBS, blocked with 3% BSA for 30 min at room temperature and subsequently incubated for 2 h with an E-cadherin monoclonal antibody (1:250 dilution, BD Biosciences). The Alexa Fluor 488 goat anti-mouse (1:250, Invitrogen) was applied for 1h30 in the dark as a secondary antibody. Nuclei were stained with DAPI (0.1 µg/ml in PBS, Sigma-Aldrich) and coverslips were mounted on slides using Vectashield (Vector Laboratories). Images were acquired on an inverted laser scanning confocal microscope (Leica TCS SP5 II, Leica Microsystems) with a HC PL APO CS 63x/Glycerol 1.30 objective, and processed with Fiji software.

### Statistical analysis

Statistical analyses were performed using the GraphPad Prism software (version 8.0.2). The Kolmogorov–Smirnov test was applied to verify whether data followed a normal distribution. Data were analysed using unpaired Student t-test with Welch correction. For evaluation of migration features over time, we have exploited the paired Student t-test. In all statistical tests, *P* ≤ 0.05 was required for significance.

## Results

### E-cadherin variants associated with HDGC and OFC impact differently cell morphology and migration abilities *in vivo*

**Fig. 1:**
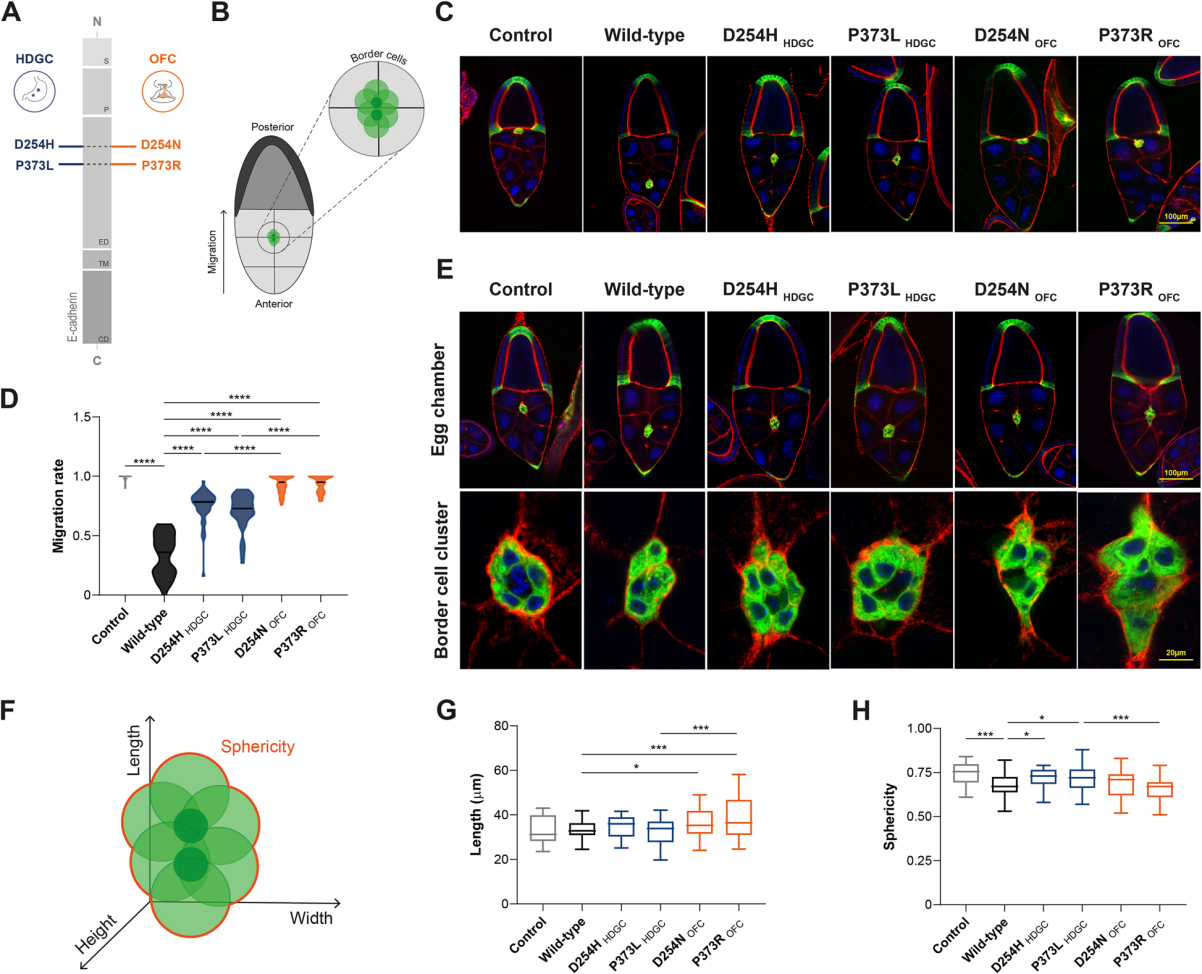
Motile and morphological features of border cells expressing HDGC and OFC variants. (**A**) Schematic representation of E-cadherin comprising the signal peptide, precursor, extracellular, transmembrane, and cytoplasmic domains. The location of D254H and P373L variants found in HDGC context, as well as D254N and P373R found in OFC patients is indicated. (**B**) Illustration of border cell migration from anterior to posterior pole of the Drosophila egg chamber. (**C**) Migration phenotypes of the different E-cadherin mutants in stage 10 egg chambers. Border cells are marked with GFP (green), phalloidin (red) stains F-actin, and the nuclei are counterstained with DAPI (blue). (**D**) Quantification of border cell migration index (*n* = 30 for each phenotype). (**E**) Images depict the morphology of border cell cluster at 50–70% of travelled distance, and corresponding close up. (**F**) Border cell cluster and morphological parameters analysed. (**G**) Average length and (**H**) sphericity of border cell clusters. Data derives from four independent experiments and * represents *P* < 0.05, ** *P* < 0.01, *** *P* < 0.001, and **** *P* < 0.0001

In order to investigate the migratory abilities yielded by *CDH1* variants associated with HDGC and OFC, we have first engineered an *in vivo* model in *Drosophila melanogaster*. In particular, wild-type hE-cadherin, D254H and P373L variants found in the HDGC context [20, 21], or D254N and P373R identified in OFC patients [22, 23] were overexpressed in border cells of the fly ovary (Fig. 1A-B) – a well-established system to study the underlying mechanisms of collective cell migration [24]. Of note, to exclude possible site-specific effects, we have selected variants affecting the same amino acid, although resulting in different disorders. With the exception of P373L, none of the studied variants were present in population databases, which argues in favour of rare alterations associating with disease. No correlation was verified between the class or predictions of amino acid alterations and the clinical manifestation (Supplementary Table 1). Likewise, all selected variants are located at the extracellular portion of the protein to prevent confounding factors related to the domain impaired (Supplementary Fig. 1A-B). We observed that expression of wild-type hE-cadherin hampers migration of border cells towards the oocyte, yielding approximately 30% of the expected distance for stage 10 egg chambers, while D254H and P373L HDGC mutants migrate 75% and 70%, respectively. Remarkably, expression of D254N and P373R OFC variants enables complete migration of border cells (Fig. 1C-D), resembling cells expressing an inert UAS-driven transgene (UAS-mCherry).

Taking advantage of this system, we have examined whether *CDH1* variants affect border cell morphology during migration (Fig. 1E-F, Supplementary Movie 1). Through 3D reconstruction of migrating cells, we were able to demonstrate that cell clusters expressing D254N and P373R OFC variants exhibited an elongated structure, reflected in significant increased length and decreased sphericity, when compared with HDGC and wild-type counterparts (Fig. 1G-H). Altogether, these results indicate that *CDH1* variants linked to OFC and HDGC display distinct morphology and migratory behaviour *in vivo*, underscoring the potential of site-specific mutants to elucidate the mechanisms that underlie divergent clinical impacts.

### OFC variants induce premature and irregular migration phenotypes *in vitro*

**Fig. 2:**
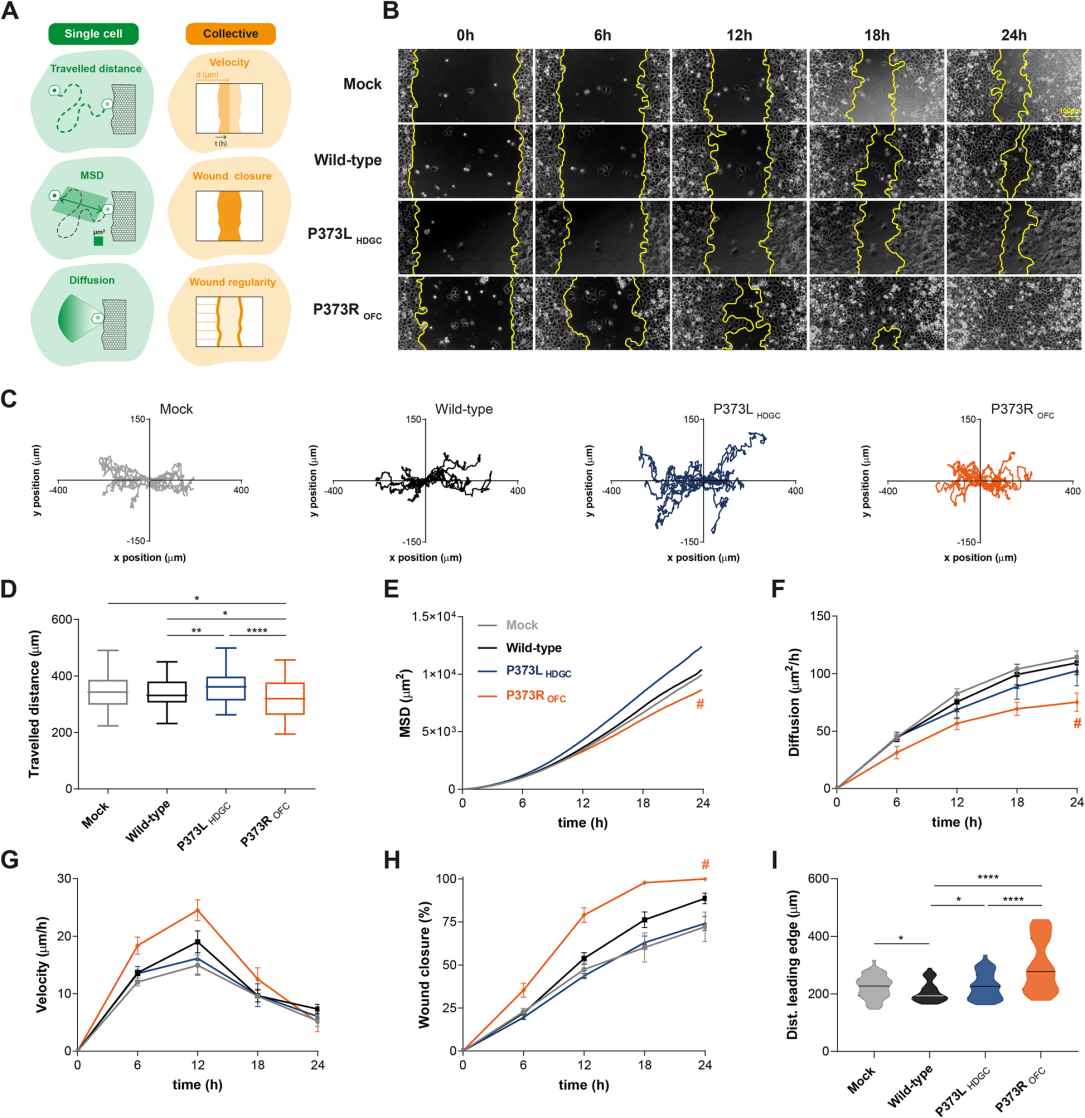
Migratory phenotypes induced by the P373L and P373R E-cadherin variants. (**A**) Scheme elucidating single cell and collective motile features extracted from images of time-lapse microscopy. (**B**) Representative migration patterns of cells expressing wild-type E-cadherin, P373L HDGC and P373R OFC variants at different time points. (**C**) Overview of cell migratory trajectories throughout 24 h. (**D**) Travelled distance, (**E**) mean-square displacement (MSD), and (**F**) diffusion rates at different time points, were calculated for individual cells. Graphs representing (**G**) speed of gap closure and (**H**) covered area over time. (**I**) Wound gap regularity determined from the distance between the initial position and the leading edge, 6 h upon device removal. Data includes results from four independent experiments. Error bars depict standard error of the mean. # represents a significant difference between P373L HDGC and P373R OFC expressing cells (*P* < 0.05)

To further explore the motile behaviour induced by OFC variants, we have established an *in vitro* model in which AGS cells (negative for cadherin expression) were transfected with vectors encoding wild-type E-cadherin and the different variants. We observed that D254 mutants lead to reduced E-cadherin levels, whereas both variants in P373 do not affect protein stability. Thus, the distinct clinical outcomes cannot be explained by changes in E-cadherin expression (Supplementary Fig. 1C-G). A comprehensive characterization of cell motility was then attained using gap closure migration assays coupled with time-lapse microscopy (Fig. 2A). We verified that E-cadherin variants associated with HDGC and OFC yield distinct migration performances and trajectory patterns. Specifically, cells expressing P373R and D254N OFC variants close the wound faster than those expressing P373L and D254H HDGC variants or the wild-type protein. Moreover, P373R and D254N single cells migrate in a direct way, in contrast to cells expressing P373L and D254H HDGC variants, which display a random migration pattern, characterized by disoriented cell movements (Fig. 2B-C and Supplementary Fig. 2). Accordingly, mean square displacement (MSD), diffusion, and overall distance travelled by individual cells are lower for OFC variants, when compared with those observed for HDGC variants (Fig. 2D-F). By analysing collective cell behaviour during wound closure, we could also detect an increased velocity of OFC cells, which is consistent with their directed movement (Fig. 2G). Interestingly, all cellular conditions reach their highest velocity at 12 h upon barrier removal. After that time, velocity decreases possibly due to a reduction in free space and to the proximity of the two leading edges. Quantitative analysis of wound closure over time revealed that cells expressing the P373R OFC variant take 8.2 h to close 50% of the gap area, while P373L HDGC cells require approximately 12 h to reach the same position (Fig. 2H). Due to enhanced migration velocities, the P373R OFC variant produced a more irregular wound leading edge, as reflected in a higher variance of the distance between the initial position and leading edge (8046.5 µm^2^ in the P373R variant, 2756.1 µm^2^ in the P373L, and 1174.5 µm^2^ in wild-type cells, Fig. 2I). Similar effects were noticed for D254H HDGC and D254N OFC variants (Supplementary Fig. 2). Thus, we conclude that OFC variants precipitate cell migration, resulting in uncoordinated tissue movements.

### OFC variants endow cells with increased adaptability in response to the ECM

**Fig. 3:**
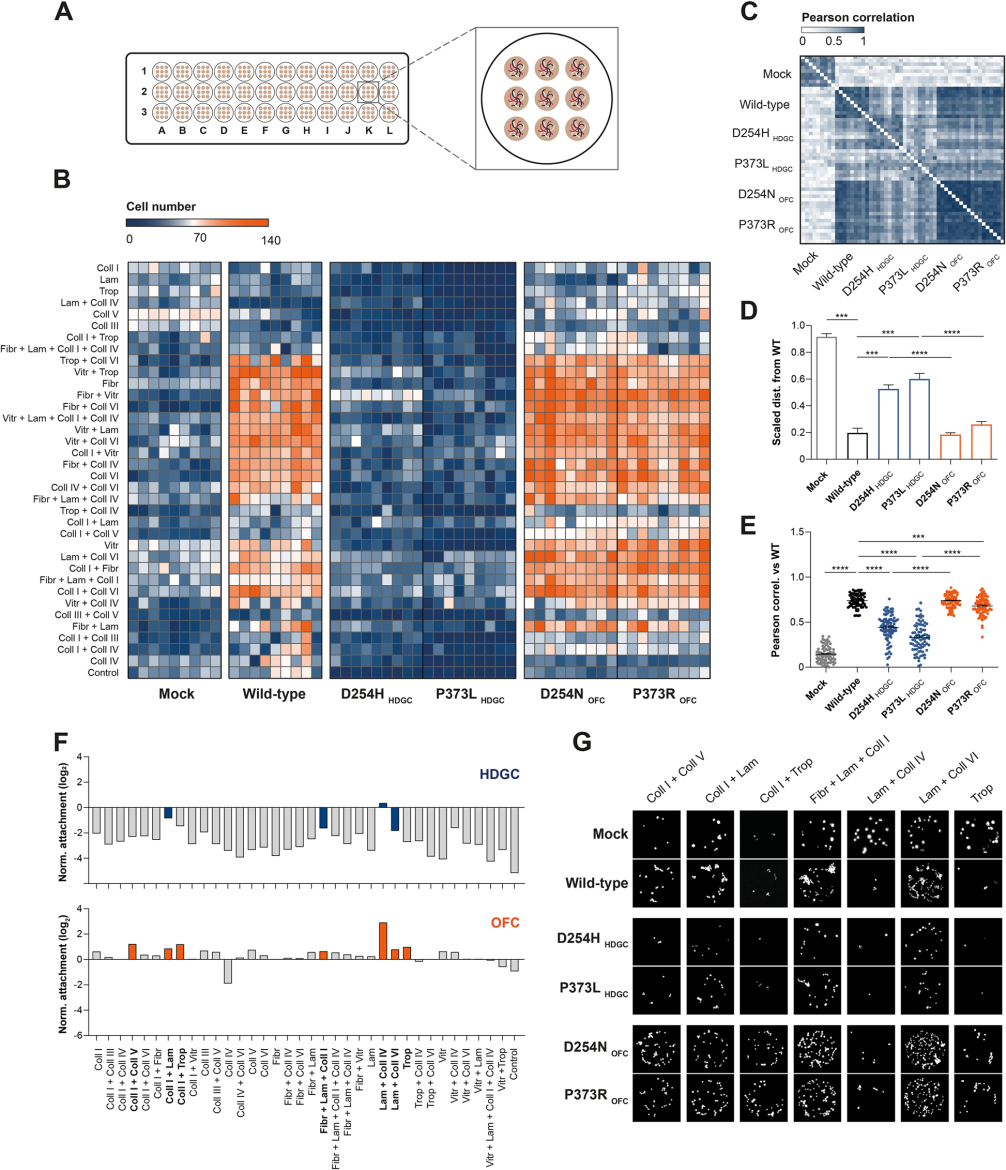
Adhesion profile associated with HDGC and OFC E-cadherin variants. (**A**) Representation of the ECM platform used to evaluate the attachment profile of HDGC and OFC variants. (**B**) Heatmap illustrating adhesive abilities of cells expressing wild-type protein or the different variants. (**C**) Matrix similarity analysis based on Pearson correlation data. (**D**) Scaled distance and (**E**) Pearson correlation between the wild-type profile and that of HDGC and OFC mutants. (**F**) Attachment of cells expressing HDGC and OFC variants normalized against the wild-type reference. ECM compositions inducing a significant difference in adhesive ability of cells are depicted in orange for OFC and in blue for HDGC. (**G**) Representative images of cell attachment in relevant ECM combinations

Given that the ECM plays a crucial role in regulating migratory behaviours [25], we addressed the impact of HDGC and OFC variants on the cell´s interplay with different matrices. For that purpose, we implemented a high-throughput ECM array with 36 combinations of ECM proteins, namely Collagen I, Collagen III, Collagen IV, Collagen V, Collagen VI, Laminin, Fibronectin, Vitronectin, and Tropoelastin (Fig. 3A). Cells transfected with vectors encoding wild-type E-cadherin or the variants found in OFC and HDGC contexts were assayed in this platform, and the number of cells attached to each ECM spot was quantified to estimate cell–matrix adhesive ability. We verified that cells expressing D254N and P373R OFC variants exhibited a greater ability to adhere to a wide panel of ECM combinations than those expressing the D254H and P373L variants identified in HDGC setting (Fig. 3B). Quantitative analysis of matrix similarity, based on Pearson correlation distance, demonstrates that OFC mutants present a significantly different matrix from that displayed by HDGC conditions (*P* < 0.0001), and a closer adhesion profile to the wild-type cells (Fig. 3C-E). Importantly, OFC variants showed a significant increase in the ability to adhere to specific compositions, namely Collagen I + Collagen V, Collagen I + Laminin, Collagen I + Tropoelastin, Fibronectin + Laminin + Collagen I, Laminin + Collagen IV, Laminin + Collagen VI, and Tropoelastin (Fig. 3F). In accordance, a high number of D254N and P373R OFC cells adhere to these ECM combinations, whereas a limited number of cells can be detected in the presence of wild-type E-cadherin, D254H, or P373L protein forms (Fig. 3G). These data suggest that OFC variants promote cell attachment to a variety of matrices, and may therefore improve cell adaptability to the surrounding niche.

### OFC and HDGC variants generate distinct transcriptomic profiles

**Fig. 4:**
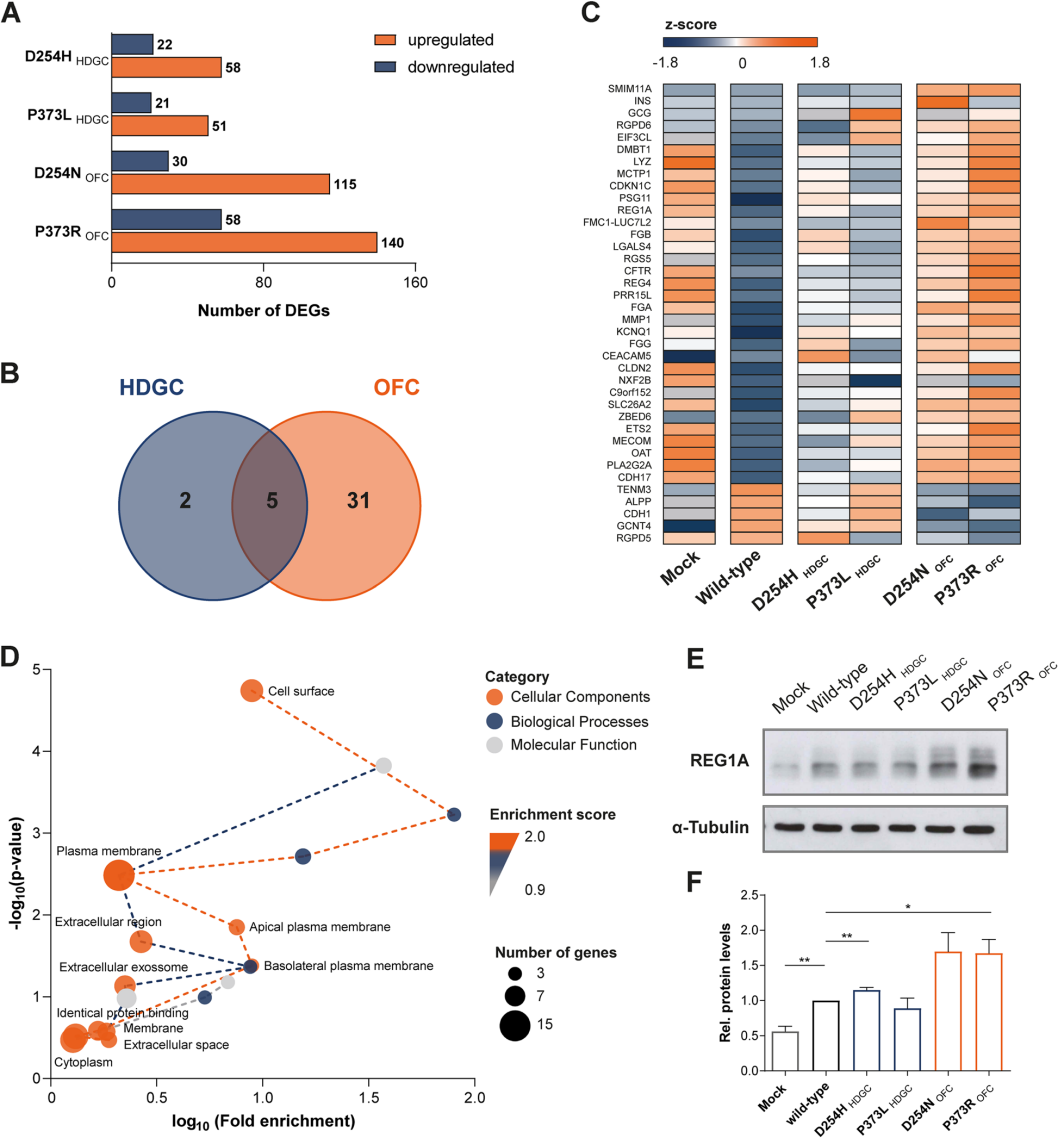
Transcriptomic analysis of cells expressing HDGC and OFC variants. (**A**) Graph showing the number of upregulated and downregulated DEGs identified in D254H, P373L, D254N, and P373R cells individually, when compared with the wild-type condition. DEGs were defined based on a fold change ≥ 2 or ≤ -2 concomitant with an FDR ≤ 0.05. (**B**) Venn diagram highlighting common and exclusive DEGs from HDGC and OFC contexts. (**C**) Heatmap representing gene expression levels of significant DEGs. Genes were sorted by fold-change. Orange indicates upregulation and blue indicates downregulation. (**D**) Functional classification and enrichment analysis reveal a network of Cellular Components, Biological Processes, and Molecular Functions associated with OFC. Dot area indicates the number of genes in each category and dashed lines represent enrichment scores. (**E**) REG1A protein level was validated by Western Blot in cells transfected with vectors encoding the different variants, as well as with wild-type protein and the empty vector (Mock). α-Tubulin was used as a loading control. (**F**) Protein quantification by densitometry in four independent experiments

To identify potential genes involved in aberrant migration and adhesive capacities of OFC mutants, we used RNA-seq technology. Analysis of mRNA from cells expressing wild-type E-cadherin or the D254H, P373L, D254N, and P373R variants revealed no bias between samples, since all conditions presented similar transcript abundance and followed a normal distribution (Supplementary Fig. 3). Wild-type expression levels were used as a reference to define up- or downregulation of genes induced by OFC and HDGC variants, with fold change ≥ 2 or ≤ -2 and an FDR ≤ 0.05 defined to screen for DEGs. The transcriptomic profile of D254N and P373R OFC variants encompasses 145 and 198 significant DEGs, respectively, whereas D254H and P373L HDGC variants result in a lower number of DEGs (Fig. 4A). However, when comparing OFC and HDGC variant groups with the wild-type profile, we could identify 46 significant DEGs, 8 of which with no functional annotation and therefore excluded. While 31 genes were exclusively altered in the OFC setting, only 2 genes were specific to the HDGC context, and 5 genes were common to OFC and HDGC phenotypes (Fig. 4B-C). Out of the 31 genes associated with OFC, 26 were found to be upregulated and 5 were downregulated (Supplementary Fig. 3D). For subsequent assessment of the biological significance of DEGs induced by OFC variants, we submitted them to gene ontology analysis, unveiling an enrichment in genes included in the Cellular Components category (Fig. 4D). In particular, Plasma Membrane and Extracellular Space were the sub-categories that stood out with the more significantly upregulated genes. Among DEGs, REG1A was selected as a strong candidate to be involved in the migratory behaviour of OFC mutants, given that it has been described to regulate cell growth, proliferation, survival, motility, and invasion in several diseases [26, 27]. Suggestive of its functional significance, we could detect increased REG1A protein levels in cells expressing D254N and P373R OFC variants, when compared with D254H and P373L HDGC variants, as well as with wild-type cells (Fig. 4E-F).

### REG1A is a key regulator of OFC migratory patterns

**Fig. 5:**
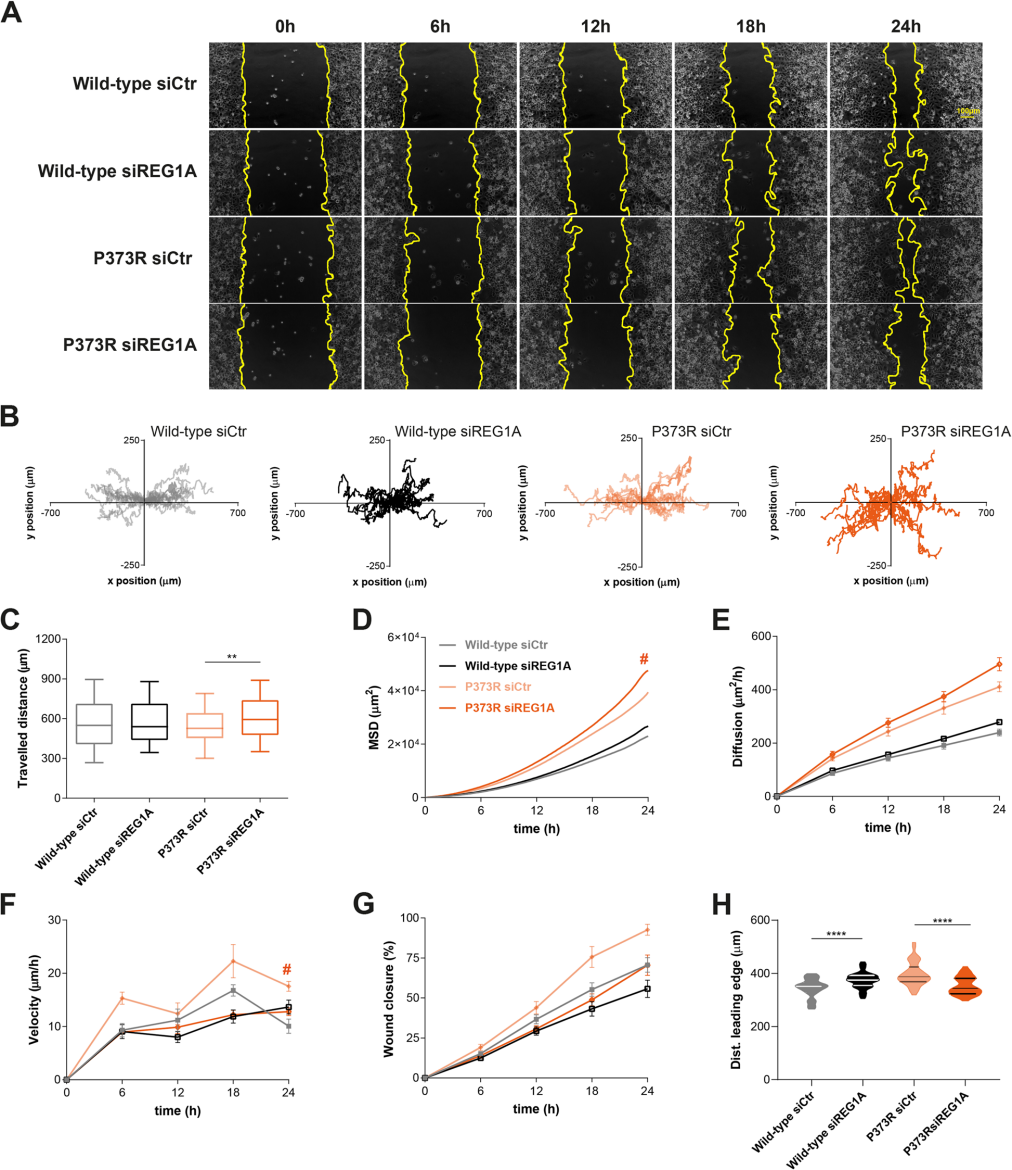
Impact of REG1A modulation in motile behaviour associated with OFC. (**A**) Representative images of the migratory phenotype of wild-type and P373R OFC cells upon treatment with siRNAs targeting REG1A. (**B**) Single cell trajectories throughout 24 h. (**C**) Overall migrating distance, (**D**) Mean-square displacement (MSD), and (**E**) diffusion rates of individual cells depleted for REG1A. (**F**) Velocity and (**G**) area of closed wound gap over time are displayed in the graphs. (**H**) Quantitative analysis of the distance between the initial position and the leading edge at 6 h upon device removal. # represents a significant difference between P373R siREG1A and P373R siRNA control cells (*P* < 0.05)

To address the role of REG1A in our model, we undertook its specific inhibition using an effective siRNA approach (Supplementary Fig. 4A-B). We observed that silencing of REG1A compromised the migratory ability of P373R OFC variant, leading to a general delay in the wound closure. A less evident impact was observed in the motility of cells depleted of REG1A and expressing wild-type E-cadherin (Fig. 5A). Moreover, upon REG1A depletion, P373R cells shifted their migration pattern from a direct to a disoriented trajectory, as confirmed by increased MSD and diffusion rates. Accordingly, the overall travelled distance by REG1A depleted P373R cells was higher than that achieved by cells treated with siRNA control (Fig. 5B-E). Concerning wound closure, we observed that P373R siRNA control cells take around 13 h to close 50% of the gap, whereas P373R siREG1A cells require approximately 18 h, which is consistent with the lower velocities observed for the latest (Fig. 5F-G). Notably, the leading edge of migrating P373R siREG1A cells presents a more regular structure when compared to the controls: variance of distance between the initial position and the leading edge was 1112.7 µm^2^ for REG1A silenced P373R cells and 1941.9 µm^2^ for the corresponding P373R siRNA control cells (Fig. 5H). Comparable data was obtained in cells expressing the D254N OFC variant upon modulation of REG1A (Supplementary Fig. 4C-J). These data indicate that REG1A is determinant for the aberrant migratory behaviour of cells expressing OFC variants.

## Discussion

In this work, we aimed to disclose the critical signalling events that are responsible for the abnormal lip/palate closure in E-cadherin germline mutation carriers. It is well established that craniofacial development is a highly intricate process, involving both spatial and temporal coordination of cell adhesion, migration, growth, and differentiation [28]. Disruption of this regulated interplay, through genetic variations and environmental factors, and their potential interactions, can result in structural birth defects, namely OFC [29, 30]. In this scope, genes associated with Bone Morphogenetic Proteins (BMPs) [31], Fibroblast Growth Factors (FGFs) [32], Sonic Hedgehog (SHH) [33], and Wnt [34] pathways are already known to influence midfacial growth and/or lip fusion. Only recently, *CDH1* germline alterations have been proposed as causative of OFC in both HDGC-dependent and -independent contexts [5, 6, 22, 35]. However, there has been little research on how mutations in this gene can trigger strikingly different clinical manifestations.

To address this unmet question, we have studied two pairs of E-cadherin missense variants located at the extracellular domain of the protein. The D254H and P373L variants were described in HDGC context [20, 36], whereas D254N and P373R have been reported in OFC patients [22, 23]. In accordance to recent studies evaluating the *CDH1* spectrum of malignancies [21], no relationship was identified regarding amino acid properties and clinical presentations. Of note, we verified that while the D254 pair leads to a decrease in protein expression, the P373 pair does not affect total protein levels. As previously described for other variants affecting the extracellular portion of E-cadherin, these may not be recognized by mechanisms of quality control and do not undergo premature degradation [13, 37]. In such cases, mutant forms can reach the plasma membrane and be rapidly internalized due to their inability to establish homophilic interactions with neighbouring cells. It is therefore not uncommon to observe aberrant cytoplasmic accumulation along with an irregular membrane staining, or even diffuse protein distribution of E-cadherin throughout the cell [21].

Our *in vivo* model first showed that E-cadherin variants associated with OFC grant cells an elongated and ergodynamic configuration, which could be linked with less resistance and increased movement across the microenvironment. As illustrated by 3D reconstructions, cell clusters expressing OFC variants extend oriented and prominent protrusions that contribute to force generation and subsequent translocation. *In vitro* experiments further confirmed an unusual ability for cells to migrate faster and with an abnormal directionality in OFC mutants, when compared with cells expressing HDGC variants.

OFC are characterized by failed fusion of the embryonic facial prominences [38], although our results suggest that this may not be the outcome of delayed cell migration. Instead, we propose that an excessive motility performance prevents the coordinated movement of the epithelial cell sheets, generating finger-like projections and irregular leading-edges, which can ultimately result in anomalous facial structures. For normal lip and palate closure, we postulate that epithelial migration must occur in a slower manner, allowing a synchronised movement at the leading edges and thereby accurate fusion of the facial prominences and palatal shelves. On the other hand, despite that HDGC mutants display slower migration, this occurs along with erratic movement of isolated cells, corroborating the diffuse dissemination abilities observed even at early stages of tumours [3, 39].

OFC variant effects are consistent with a phenotype of mechanical advantage provided by attachment to the ECM [40]. Accordingly, we found that cells expressing OFC variants adhere to a wide range of ECM substrates, suggesting increased ability to adjust when compared to cells carrying HDGC alterations. More so, ECM combinations preferred by OFC mutants enclose structural elements for palatogenesis, namely type I, IV, V, and VI collagens [41]. Mansell and colleagues were the first to notice alterations in collagen cross-linking and turnover, as well as an overall increase in collagen content during rodent palatal development [42]. Interestingly, ECM composition and remodelling were suggested to play a role in the aetiology of CL/P [43]. An imbalance in fibronectin abundance is associated with CL/P [44–46], in line with the lower adherence of OFC mutant cells to fibronectin observed herein. On the contrary, it was recently demonstrated that gastric cancer cells bind selectively to fibronectin-enriched ECMs, where they are able to exert increased traction forces [47].

These results prompted us to investigate whether plasticity of OFC mutant cells would be related with the activation of a dedicated expression profile. Following implementation of an RNA-seq workflow, it was verified that OFC and HDGC genotypes produce distinct molecular signatures, exposing a high number of DEGs in the OFC context. In addition, the OFC group was enriched in gene sets of Plasma Membrane and Extracellular Space subclasses, corroborating the improved cell–matrix attachment observed in OFC mutants. Among significantly overexpressed candidate genes, we selected REG1A as a potential regulator of the OFC phenotype. Thus, confirmation of REG1A upregulation at the protein level motivated its modulation through siRNA assays. We found that REG1A downregulation rescues the aberrant epithelial movement produced by OFC mutants, forming a more regular and slower migration front. Moreover, individual cells exhibit a clear lack of orientation cues and undertake random trajectories. The same trend, although less striking, was observed for trajectories and velocity of wild-type cells after REG1A inhibition. This is consistent with previous work showing that REG1A overexpression was associated with invasion and poor prognosis of colorectal and bladder cancer patients, while its inhibition impaired cancer cell migration [27, 48]. In gastric carcinomas, there is limited data on the role of REG1A, despite reports of its downregulation through epigenetic methylation [49].

In agreement with the present findings, other studies have already proposed deregulated adhesion and migration programs as critical determinants of OFC pathogenesis [50, 51]. Nonetheless, their significance was not addressed at the cellular level, limiting interpretation and translation of data into the clinics.

## Conclusion

This is the first evidence that E-cadherin synergizes with a specific signalling pathway impacting cell migratory behaviour in the context of congenital malformations. We have shown that OFC E-cadherin variants lead to abnormal tissue shaping and movement in a REG1A-dependent manner. Ultimately, we demonstrate the potential of site-specific mutant cell lines and fly models for the discovery of cellular mechanisms involved in disease intricacies. The significance of this study goes beyond the understanding of the molecular networks underlying OFC and HDGC. In the future, genetic counselling of *CDH1* variant carriers could benefit from variant classification pipelines including the assessment of migration features through *in vitro* assays. This will be crucial to identify those at risk of gastric cancer and those that could have a neonate affected by congenital anomalies, such as OFC.

### Supplementary Information

**Additional file1:** **Supplementary Movies 1.** Modelling of border cell clusters expressing the D254H, P373L, D254N, and P373R E-cadherin variants, or the wild-type form. 3D reconstruction of cluster morphology using Imaris Software.

**Additional file 2.**

**Additional file 3.**

**Additional file 4.**

**Additional file 5.**

**Additional file 6.**

**Additional file 7:** **Supplementary Figure 1.** E-cadherin structure, expression and localization in HDGC and OFC cell mutants. (A) 3D visualization of E-cadherin structure with Mol* Viewer, highlighting the D254 and P373 positions. (B) Impact of D254H, P373L, D254N and P373R variants in protein conformation. (C) Protein levels were analysed by Western Blot in (C-D) CHO and (E-F) AGS cells transfected with vectors encoding wild-type E-cadherin and the different variants, or the empty vector (Mock) as a control. α-Tubulin was used as a loading reference. Band intensity was quantified and normalized against wild-type cells. (G) Immunofluorescence was applied to evaluate protein localization. E-cadherin is shown in green and nuclei were counterstained with DAPI (blue). Graphs show quantification of signal intensities along contiguous cells (internuclear profiles). Position 1 and 100 correspond to the geometric centers of nucleus 1 and nucleus 2, respectively. Position 50 represents the plasma membrane.

**Additional file 8:** **Supplementary Figure 2. **Migratory phenotypes generated by D254H and D254N E-cadherin variants. (A) Representative images of time-lapse microscopy illustrating the migration pattern of cells expressing D254H HDGC and D254N OFC variants at different time points. (B) Migratory trajectories of individual cells during 24 hours. (C) Total travelled distance, (D) Mean-square displacement (MSD), and (E) cell spreading rate at different time points are displayed. (F) Graphs represent the speed of monolayer movement, (G) percentage of wound field over time, and (H) leading edge regularity, 6h upon device removal. # represents a significant difference between D254H HDGC and D254N OFC expressing cells (*P*<0.05).

**Additional file 9:** **Supplementary Figure 3. **RNA sequencing data analysis for D254H, P373L, D254N, and P373R E-cadherin mutants. (A) Abundance distribution of all identified transcripts in transcripts per million (TPM), and (B) density distribution of each RNA sample. (C) Principal Component Analysis (PCA) showing the transcriptomic profile of cells transfected with the empty vector (Mock), wild-type E-cadherin or the different variants. (D) Unique up- and downregulated DEGs from the OFC setting.

**Additional file 10:** **Supplementary Figure 4**. Effects of REG1A depletion in motility of D254N OFC cells. (A) Specific inhibition of REG1A was performed on cells stably transfected with D254N and P373R E-cadherin. REG1A levels were analysed by Western blot. α-Tubulin was used as a loading control. (B) Band intensity was quantified and normalized to wild-type cells treated with non-targeting siRNA. (C) Time-lapse microscopy showing the migration pattern of cells upon REG1A silencing. (D) Trajectories resulting from single cell tracking throughout 24 hours. Analysis of individual motile cells regarding (E) travelled distance, (F) mean-square displacement (MSD), and (G) diffusion rate. (H) Speed of gap closure, (I) area of wound field, and (J) wound gap regularity are displayed in the graphs. # represents a significant difference between D254N siREG1A and D254N siRNA control cells (*P*<0.05).

**Additional file 11.**

**Additional file 12.**

## Data Availability

Raw sequencing data has been deposited at the NCBI Sequence Read Archive (PRJNA1051159). https://dataview.ncbi.nlm.nih.gov/object/PRJNA1051159?reviewer=jslcla2aatah6ve66rtoqca9d
