# Supplementary figures and images for: E-cadherin variants associated with oral facial clefts trigger aberrant cell motility in a REG1A-dependent manner
Source: Cell Commun Signal. 2024 Feb 27;22:152. doi: 10.1186/s12964-024-01532-x (PMC10898076; doi:10.1186/s12964-024-01532-x)

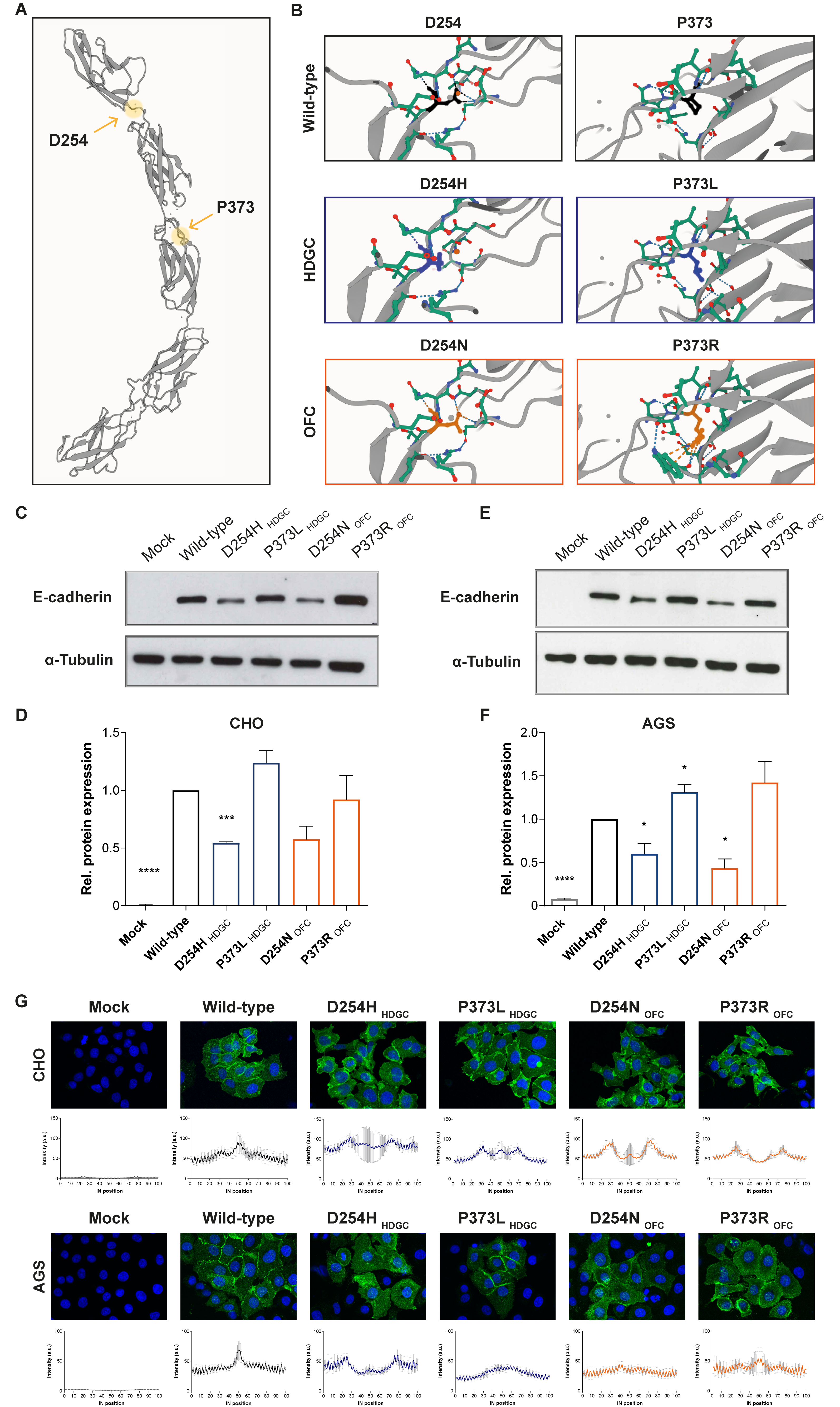

Supplement: Supplementary file 7 — Additional file 7: Supplementary Figure 1. E-cadherin structure, expression and localization in HDGC and OFC cell mutants. (A) 3D visualization of E-cadherin structure with Mol* Viewer, highlighting the D254 and P373 positions. (B) Impact of D254H, P373L, D254N and P373R variants in protein conformation. (C) Protein levels were analysed by Western Blot in (C-D) CHO and (E-F) AGS cells transfected with vectors encoding wild-type E-cadherin and the different variants, or the empty vector (Mock) as a control. α-Tubulin was used as a loading reference. Band intensity was quantified and normalized against wild-type cells. (G) Immunofluorescence was applied to evaluate protein localization. E-cadherin is shown in green and nuclei were counterstained with DAPI (blue). Graphs show quantification of signal intensities along contiguous cells (internuclear profiles). Position 1 and 100 correspond to the geometric centers of nucleus 1 and nucleus 2, respectively. Position 50 represents the plasma membrane. [file 12964_2024_1532_MOESM7_ESM.tif]

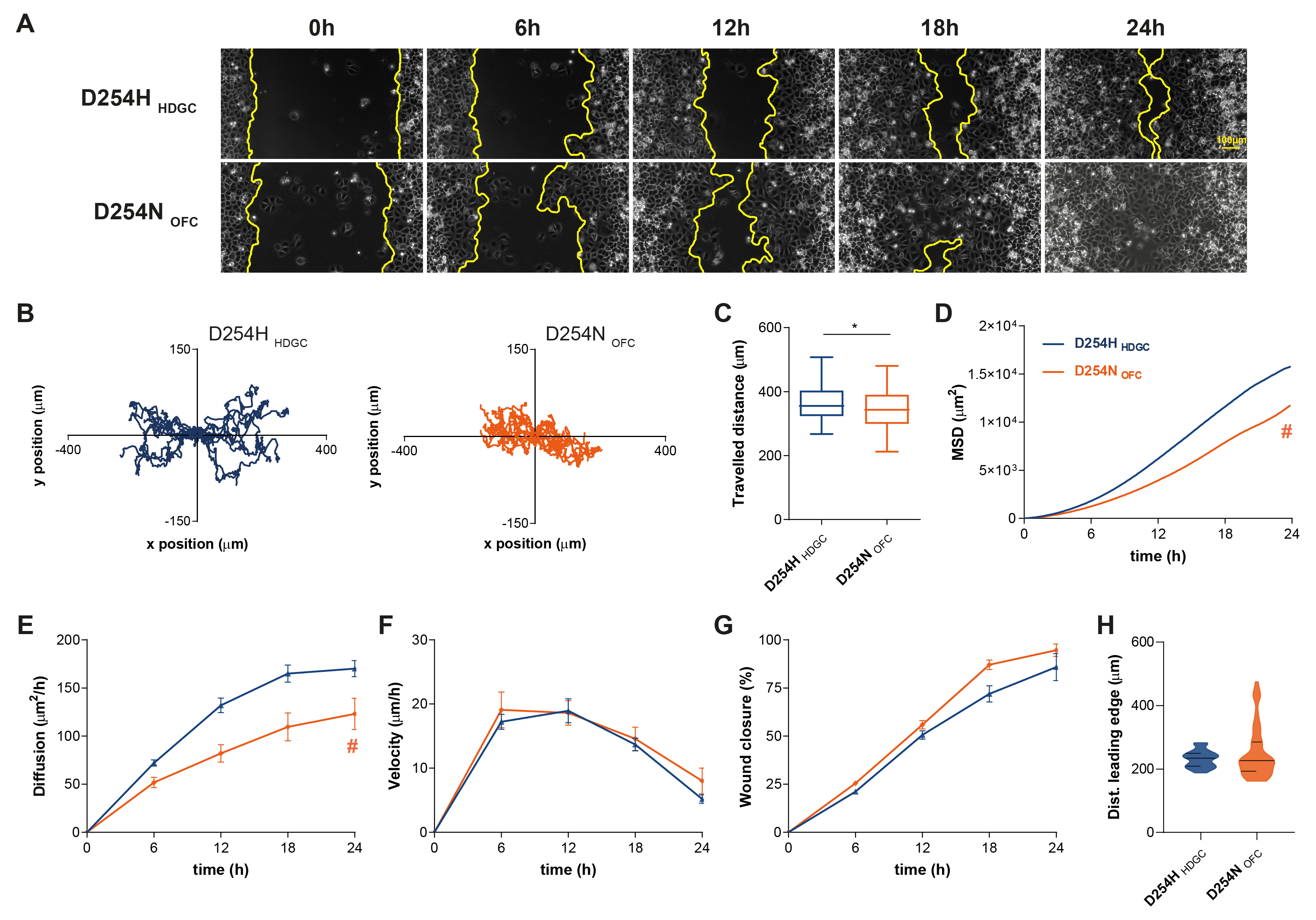

Supplement: Supplementary file 8 — Additional file 8: Supplementary Figure 2. Migratory phenotypes generated by D254H and D254N E-cadherin variants. (A) Representative images of time-lapse microscopy illustrating the migration pattern of cells expressing D254H HDGC and D254N OFC variants at different time points. (B) Migratory trajectories of individual cells during 24 hours. (C) Total travelled distance, (D) Mean-square displacement (MSD), and (E) cell spreading rate at different time points are displayed. (F) Graphs represent the speed of monolayer movement, (G) percentage of wound field over time, and (H) leading edge regularity, 6h upon device removal. # represents a significant difference between D254H HDGC and D254N OFC expressing cells (P<0.05). [file 12964_2024_1532_MOESM8_ESM.tif]

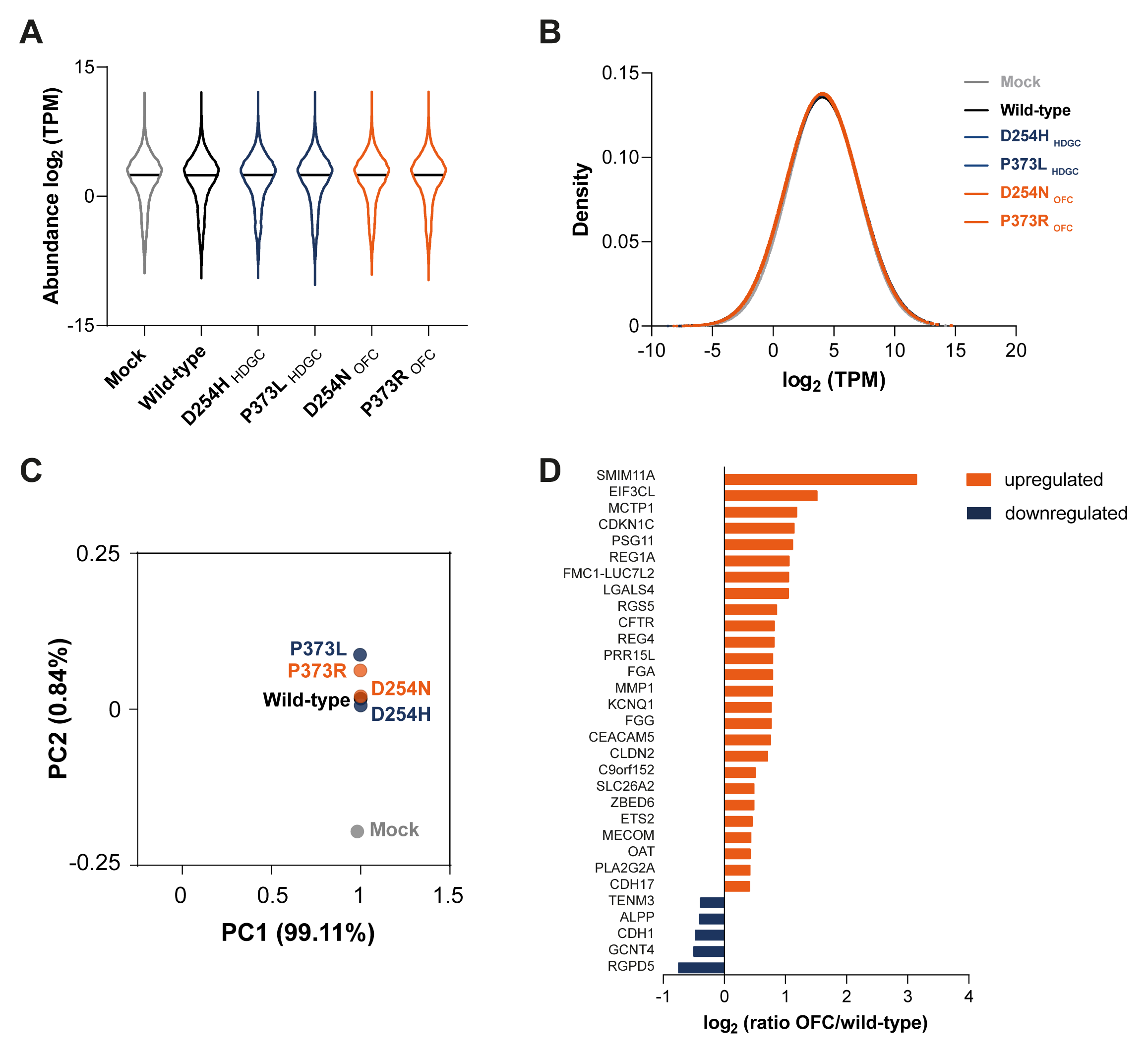

Supplement: Supplementary file 9 — Additional file 9: Supplementary Figure 3. RNA sequencing data analysis for D254H, P373L, D254N, and P373R E-cadherin mutants. (A) Abundance distribution of all identified transcripts in transcripts per million (TPM), and (B) density distribution of each RNA sample. (C) Principal Component Analysis (PCA) showing the transcriptomic profile of cells transfected with the empty vector (Mock), wild-type E-cadherin or the different variants. (D) Unique up- and downregulated DEGs from the OFC setting. [file 12964_2024_1532_MOESM9_ESM.tif]

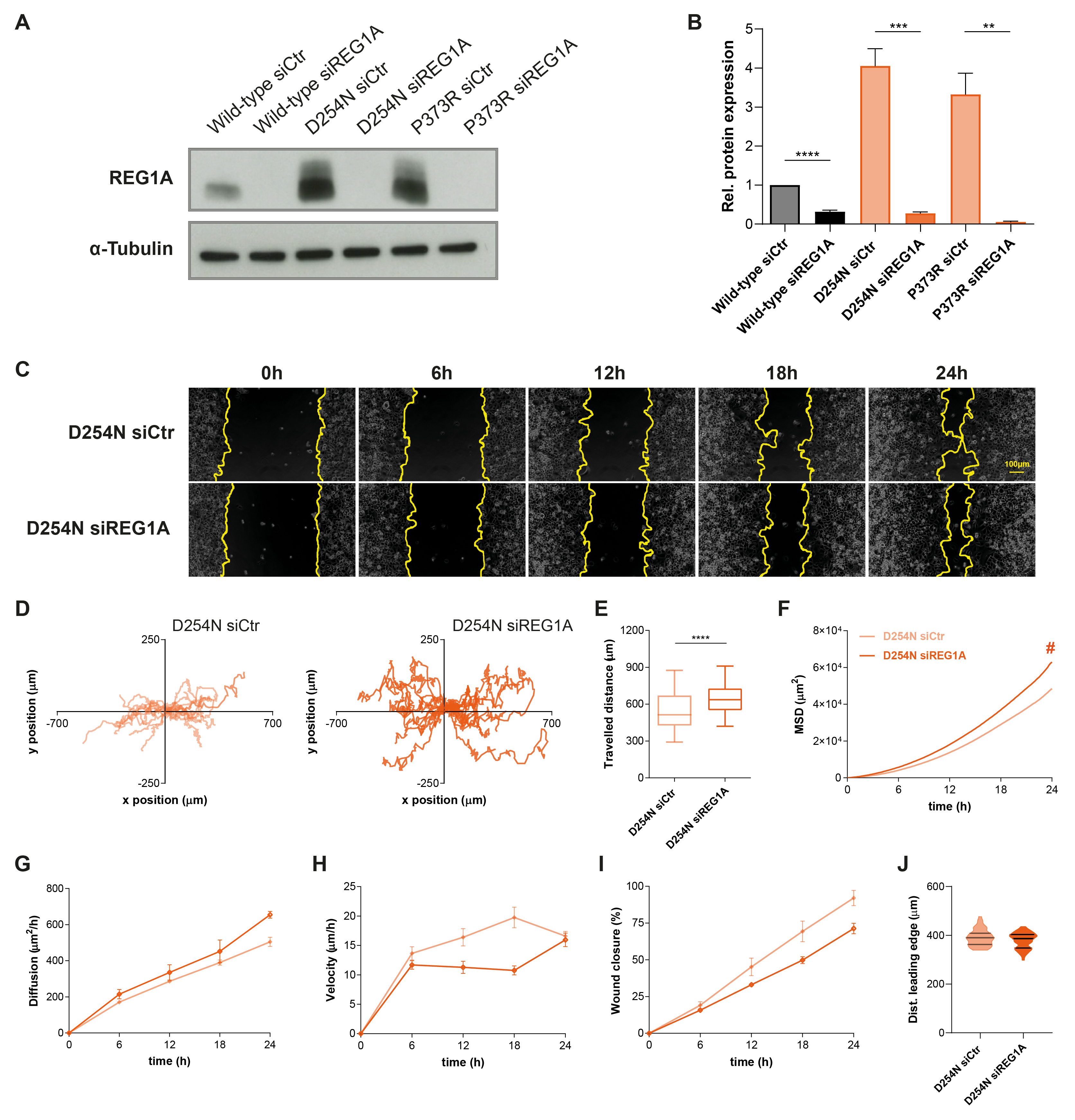

Supplement: Supplementary file 10 — Additional file 10: Supplementary Figure 4. Effects of REG1A depletion in motility of D254N OFC cells. (A) Specific inhibition of REG1A was performed on cells stably transfected with D254N and P373R E-cadherin. REG1A levels were analysed by Western blot. α-Tubulin was used as a loading control. (B) Band intensity was quantified and normalized to wild-type cells treated with non-targeting siRNA. (C) Time-lapse microscopy showing the migration pattern of cells upon REG1A silencing. (D) Trajectories resulting from single cell tracking throughout 24 hours. Analysis of individual motile cells regarding (E) travelled distance, (F) mean-square displacement (MSD), and (G) diffusion rate. (H) Speed of gap closure, (I) area of wound field, and (J) wound gap regularity are displayed in the graphs. # represents a significant difference between D254N siREG1A and D254N siRNA control cells (P<0.05). [file 12964_2024_1532_MOESM10_ESM.tif]
