# Supplementary material for: E-cadherin variants associated with oral facial clefts trigger aberrant cell motility in a REG1A-dependent manner
Source: Cell Commun Signal. 2024 Feb 27;22:152. doi: 10.1186/s12964-024-01532-x (PMC10898076; doi:10.1186/s12964-024-01532-x)
Supplement: Supplementary file 11 — Additional file 11. [file 12964_2024_1532_MOESM11_ESM.docx]

**Supplementary Table 1. Amino acid description and *in silico* analysis of *CDH1* variants.** For each variant, the type of original and substituted amino acid is indicated. Potential impact of variants was evaluated using SIFT, PolyPhen2, Provean, and FoldX, which consider sequence homology, physical properties of amino acids, and protein 3D structure. Variants generating a score below 0.05 were classified as damaging by SIFT; Provean score equal or below -2.5 suggests a deleterious variant; variants leading to a ∆∆G>0.8 kcal/mol calculated with FoldX are associated to protein destabilization.

|  | | Amino acid classes | | SIFT | | PolyPhen2 | | Provean | | FoldX | |
| --- | --- | --- | --- | --- | --- | --- | --- | --- | --- | --- | --- |
| Variant | **Phenotype** | **Original** | **Altered** | **Prediction** | **Score** | **Prediction** | **Score** | **Prediction** | **Score** | **Prediction** | **Score** |
| D254H | HDGC | Negatively charged (Asp) | Positively charged (His) | Damaging | 0.000 | Probably damaging | 1.000 | Deleterious | -6.47 | Structural impact | 2.038 |
| D254N | OFC | Negatively charged (Asp) | Polar, uncharged (Asn) | Damaging | 0.020 | Probably damaging | 1.000 | Deleterious | -4.62 | Neutral | -0.812 |
| P373L | HDGC | Nonpolar (Pro) | Nonpolar (Leu) | Damaging | 0.001 | Probably Damaging | 1.000 | Deleterious | -9.53 | Structural impact | 1.103 |
| P373R | OFC | Nonpolar (Pro) | Positively charged (Arg) | Damaging | 0.001 | Probably Damaging | 1.000 | Deleterious | -8.58 | Structural impact | 3.537 |
