# Supplementary material for: E-cadherin variants associated with oral facial clefts trigger aberrant cell motility in a REG1A-dependent manner
Source: Cell Commun Signal. 2024 Feb 27;22:152. doi: 10.1186/s12964-024-01532-x (PMC10898076; doi:10.1186/s12964-024-01532-x)

Supplementary file 1

Uncropped Blot images from Figure 4E

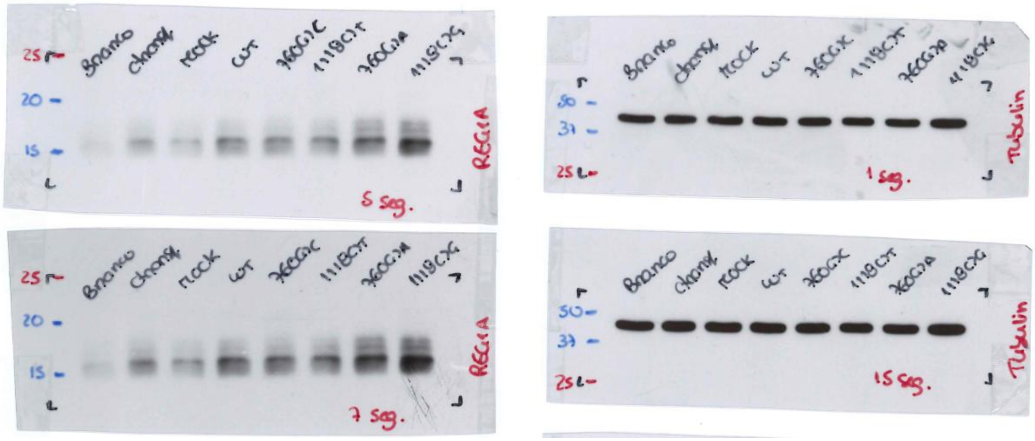

Uncropped Blot images from Supplementary Figure 1C

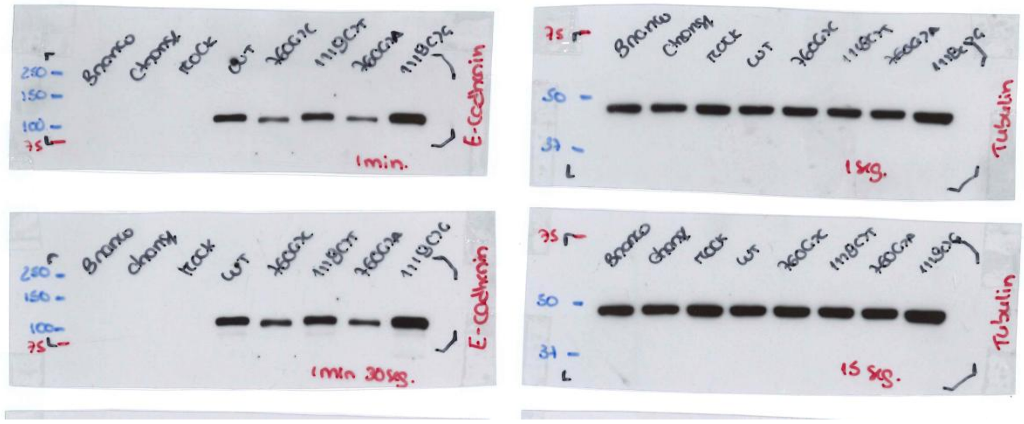

Uncropped Blot images from Supplementary Figure 1E

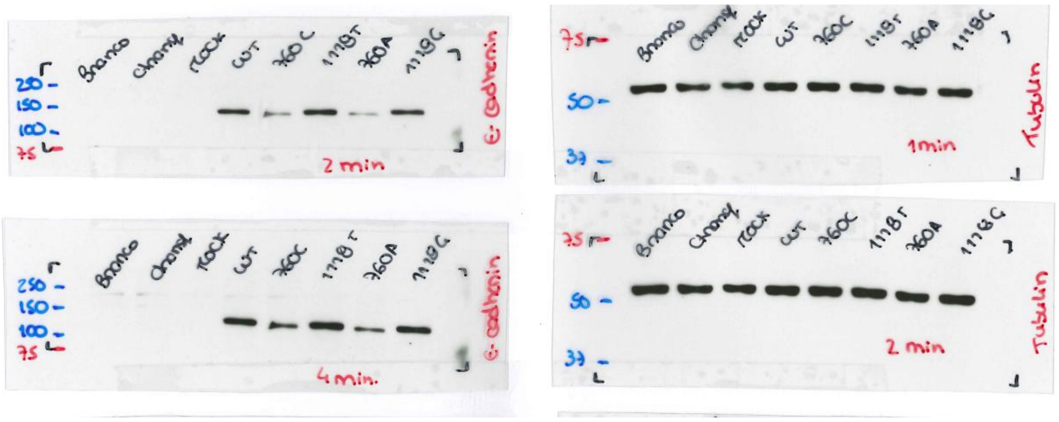

Uncropped Blot images from Supplementary Figure 4A

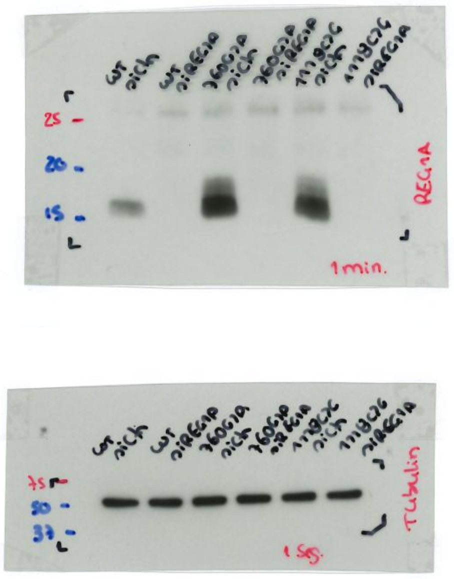

Supplement: Supplementary file 12 — Additional file 12. [file 12964_2024_1532_MOESM12_ESM.pdf]
